# Cross-cultural adaptation, reliability and validity of the Turkish version of the spine functional index

**DOI:** 10.1186/s12955-015-0219-3

**Published:** 2015-02-27

**Authors:** Eda Tonga, Charles Philip Gabel, Sedef Karayazgan, Antonio I Cuesta-Vargas

**Affiliations:** Department of Physiotherapy and Rehabilitation, Baskent University, Faculty of Health Sciences, Ankara, Turkey; Faculty of Science, Centre for Healthy Activities, Sport and Exercise, University of the Sunshine Coast, Sunshine Coast, QLD Australia; Department of Ergotherapy, Hacettepe University, Faculty of Health Sciences, Ankara, Turkey; Department of Physiotherapy, Faculty of Health Science, University of Malaga, Malaga, Spain; School of Clinical Science, Faculty of Health, Queensland University of Technology, Kelvin Grove, Australia

**Keywords:** Spine outcome, Turkish, Validity, Reliability, Factor analysis

## Abstract

**Background:**

The Spine Functional Index (SFI) is a patient reported outcome measure with sound clinimetric properties and clinical viability for the determination of whole-spine impairment. To date, no validated Turkish version is available. The purpose of this study is to cross-culturally adapted the SFI for Turkish-speaking patients (SFI-Tk) and determine the psychometric properties of reliability, validity and factor structure in a Turkish population with spine musculoskeletal disorders.

**Methods:**

The SFI English version was culturally adapted and translated into Turkish using a double forward and backward method according to established guidelines. Patients (n = 285, cervical = l29, lumbar = 151, cervical and lumbar region = 5, 73% female, age 45 ± 1) with spine musculoskeletal disorders completed the SFI-Tk at baseline and after a seven day period for test-retest reliability. For criterion validity the Turkish version of the Functional Rating Index (FRI) was used plus the Neck Disability Index (NDI) for cervical patients and the Oswestry Disability Index (ODI) for back patients. Additional psychometric properties were determined for internal consistency (Chronbach’s α), criterion validity and factor structure.

**Results:**

There was a high degree of internal consistency (α = 0.85, item range 0.80-0.88) and test-retest reliability (r = 0.93, item range = 0.75-0.95). The factor analysis demonstrated a one-factor solution explaining 24.2% of total variance. Criterion validity with the ODI was high (r = 0.71, p < 0.001) while the FRI and NDI were fair (r = 0.52 and r = 0.58, respectively). The SFI-Tk showed no missing responses with the ‘half-mark’ option used in 11.75% of total responses by 77.9% of participants. Measurement error from SEM and MDC_90_ were respectively 2.96% and 7.12%.

**Conclusions:**

The SFI-Tk demonstrated a one-factor solution and is a reliable and valid instrument. The SFI-Tk consists of simple and easily understood wording and may be used to assess spine region musculoskeletal disorders in Turkish speaking patients.

## Background

Patient reported outcome (PRO) instruments are generally used for assessing the patients’ functional status, activity limitation, participation restriction, quality of life and pain level [1]. Spinal musculoskeletal problems are well-recognized with an associated functional limitation that may be considered as a major cause of disability. The most common spinal regions studied are the lumbar and cervical, predominantly due to their symptomatic prevalence in the general population [2-5]. Patients with spinal musculoskeletal disorders are commonly measured with objective physical assessments including range of motion, muscle strength, neurologic tests and so forth. The use of PRO instruments is important for determining a patient’s perceptions of their general health and conditions that affect them. Patients with spine problems often experience difficulties in daily function and these problems are generally assessed by means of PRO instruments [6,7]. The PROs that assess the spine remain distinctly divided into back and neck with several developed for assessing these sub-regions. Their importance as indicators of the effectiveness of interventions and the subsequent outcomes of clinical trials are well recognized [6-10]. However there is no consensus on what is the optimal spinal PRO and the instruments available for assessing the spine as a single kinetic chain are limited [8]. Researchers and clinicians are consequently confronted with many different PROs to assess their patients with spinal disorders [6-10].

Three commonly used questionnaires for assessing low back disability are the Roland-Morris Disability Questionnaire (RMQ), the Oswestry Disability Index (ODI) and the Quebec Back Pain Disability Scale. However, only the RMQ and ODI were translated and validated for the Turkish speaking population [11-13]. The Neck Disability Index (NDI) is the most widely used instrument to assess the functional status of patient’s with a problematic neck [14-16]. The Turkish version is available and used in clinical practice and research [17,18]. Another measurement option is the generic PRO such as the Short Form 36 Health Survey (SF-36) or its derivatives the SF-12 and SF-8, along with the EuroQol.

By contrast few whole-spine PROs are available or recommended due to documented problems with either or both the psychometric and practical characteristics. Whole-spine PROs assess the spine from the cervical to lumbar regions as a continuous single kinetic-chain. A total of five PROs purport validity for the whole-spine [8] with the Functional Rating Index (FRI), the most commonly advocated due to its preferred administrative practicality and level of independent research on comparative clinimetric properties for both back and neck conditions [8,19]. In Turkey, clinicians and researchers commonly choose the ODI for back patients and the NDI for neck patients. But there is a gap in the knowledge base as to whether there is a clinimetrically sound option that covers both areas and can also serve patients with either whole-spine problems or when there is a need to compare different patients with back or neck problems. To date there is only one research study that investigated reliability and validity of the FRI in a Turkish population and that used older people with low back pain only. There has been no study that had adapted the FRI culturally and linguistically to Turkish. The few Turkish researchers who have used the FRI in their studies have done so with a translated but not culturally and linguistically adapted version. Consequently the FRI cannot be used as the primary assessment tool in a study, only as a supporting and secondary outcome measure.

A recently developed whole-spine PRO, the Spine Functional Index (SFI), has addressed the limitations of existing whole-spine PROs. The SFI was also shown to have preferred clinimetric properties to the FRI to which it was compared concurrently in a prospective trial [8]. Consequently, the aim of this study was to cross-culturally adapt the SFI for Turkish-speaking patients (SFI-Tk) and to determine the psychometric properties of validity, reliability, internal consistency, measurement error and factor structure in patients with spine musculoskeletal problems that affected any or all of the regions of the neck, back and/or low back.

## Materials and methods

### Subjects

Subject inclusion criteria were an age minimum of 18 years, symptoms duration of ˃12 weeks, providing a chronic population, and being referred by a medical practitioner to the Baskent University Physical Therapy Clinic with a diagnosis of a musculoskeletal spine condition or symptoms.

Exclusion criteria were an inability to read Turkish or respond to the questionnaires, an inflammatory condition, recent surgery, pregnancy, infectious disease, neurological diseases, cancer or other systemic diseases with possible effects on spine function. The study was approved by Baskent University Non-Interventional Clinical Researches Ethics committee.

### Procedure

Data was collected at baseline by a physiotherapist on the day of initial attendance. All participants were informed of the study’s details and signed informed consent was obtained. All patients concurrently completed the SFI-Tk and FRI-Tk where the latter served for the determination of criterion validity of the whole spine. In addition patients with back or low back problems also completed the ODI-Tk and patients with neck problems completed the NDI-Tk. These latter two instruments respectively gave an independent criterion validation for the back and neck sub-regions. Patients were asked to repeat the SFI for test-retest reliability on subsequent attendance after a seven day period of non-treatment.

### Questionnaires

The SFI is a single page 25-item PRO with a three-point response option for each item of ‘Yes’ , ‘Partly’ or ‘No’ [8] completed in reference to the patient’s functional status ‘over the last few days’. The scores from the 25 items are added, this score is then multiplied by four and subtracted from 100 to generate a 0-100% score (0% = maximum limitation or functional loss and 100% = no disability, normal or pre-injury status). Up to two missing responses are permitted [8].

The original FRI [19] contains 10 items with each rated on a five-point Likert scale that incorporated both visual and descriptive response options in reference to the patient’s functional status ‘today’. The original instrument was a two page PRO and a format modified single-page version was used in this study. Five FRI items are common to the ODI and NDI with the remaining five items being three additional ODI items, one NDI item and a new ‘pain’ item. The FRI raw score is multiplied by 2.5 to generate a 0-100% score (0% = no disability and 100% = maximum disability). One missing response is permitted.

The ODI [20] consists of ten items and is completed in reference to the patient’s functional status ‘today’. Each item contains six statements on a 0–5 points scale. The maximum possible score is 50 with the total score converted to a percentage by doubling the value. The subjective categorization of status is represented as follows: 0-20% indicates minimal disability, 21-40% moderate disability, 41- 60% severe disability, 61-80% crippled and 81-100% total incapacitation [12,20].

The Neck Disability Index (NDI) is derived from the ODI and contains ten items in reference to the patient’s functional status ‘today’. Seven items assess daily activities, two assess pain and one is related to concentration. Each question has six descriptive response options on a 0–5 points scale. The maximum possible score is 50 with the total score converted to a percentage scale by doubling the raw score. The NDI raw scores can be used to categorize disability: no disability (0 to 4), mild (5 to 14), moderate (15 to 24), severe (25 to 34), and complete disability (greater than 34) [17,21].

### Translation and cross-cultural adaptation

**Figure 1:**
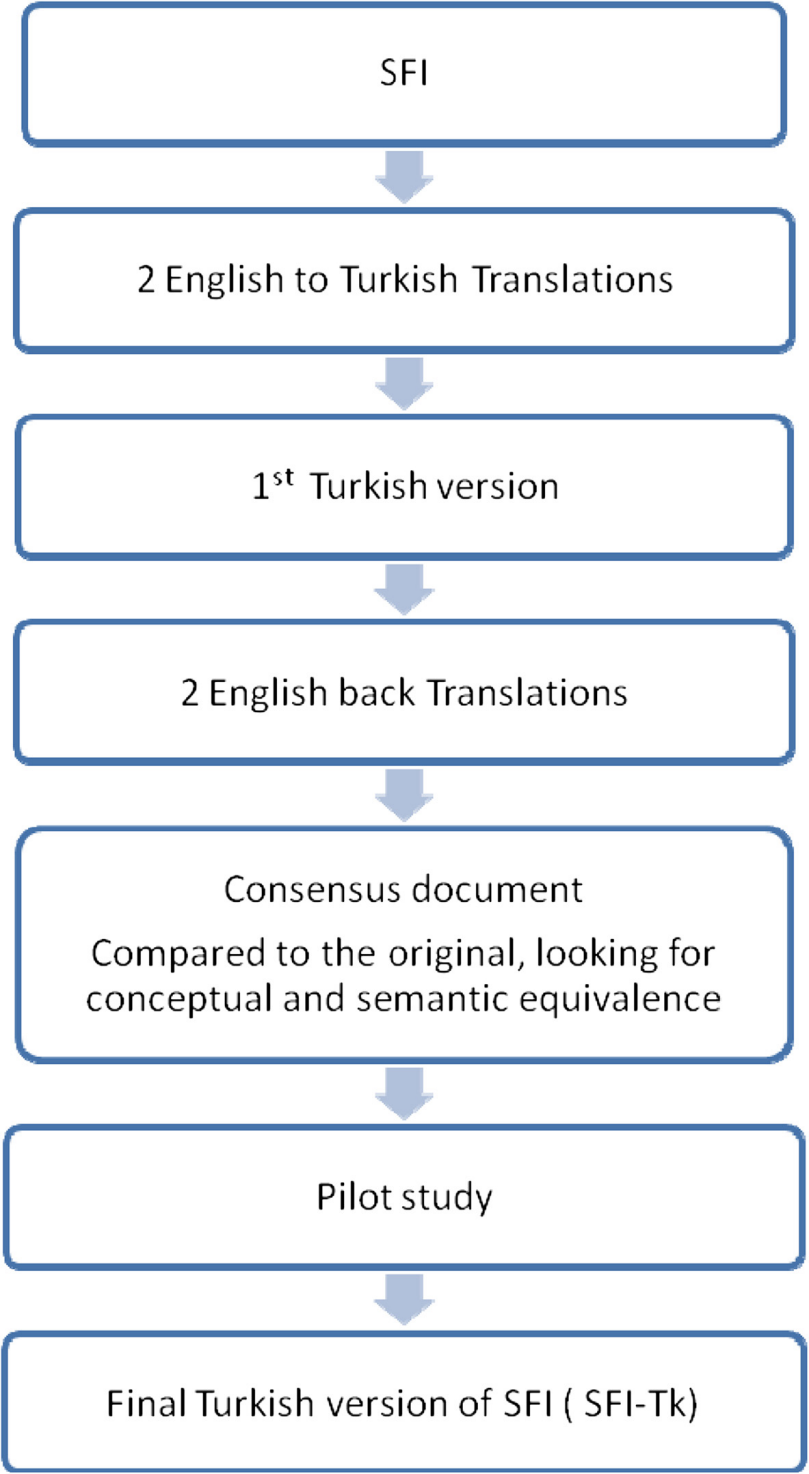
**Flowchart of the translation of the Spine Functional Index (SFI) from English to Turkish.**

Translation of the SFI was performed using a double forward and backward method [22] and conformed to the COSMIN recommendations [23]. This also provided an initial indication of face and content validity. Two Turkish native-language translators performed forward translation independently. This allowed detection of errors and divergent interpretations of items with ambiguous meanings. To improve idiomatic and conceptual (rather than literal) equivalence and improve reliability, one translator had knowledge of the questionnaires concepts and the study’s purpose. This enabled any unexpected meanings in the original tool to be recognized. Back translation was performed blindly and independently by two English native-language speakers with the final versions compared to the original version for inconsistencies and to provide a final consensus version (Figure 1).

## Statistics

*Descriptive analyses* were applied to calculate means and standard deviations of the demographic variables (Table 1). *Distribution and normality* were determined by the one-sample Kolmogorov-Smirnov tests (significance >0.05). Gender differences in the item responses were determined by one-way analysis of variance (ANOVA). *Construct validity and factor structure* were determined from maximum likelihood extraction (MLE) with the *a-priori* extraction requirements being satisfaction of three criteria: screeplot inflection, Eigenvalue >1.0 and variance >10% [24,25]. The recommended minimum ratio of ten participants-per-item was satisfied [26]. *Exploratory factor analysis* indicated a single factor structure was likely, therefore more >250 participants were required [27]. The *internal consistency* was determined from Cronbach's α coefficient [28]. *Criterion validity* was determined through the concurrent use of all PRO instruments (FRI-Tk, NDI-Tk, ODI-Tk and SFI-Tk). The Pearson’s r correlation coefficient used the criteria of poor (r < 0.49), fair (r = 0.50-0.74) and strong (r > 0.75) [29].

**Table 1:** **Demographic characteristics and frequency of diagnosis of the study population**

***Characteristic study population***	***Cases (%) 285***
**Male**	78 (27%)
**Female**	207 (73%)
**Age (years)**	45±1
***Sub-region***	
**Cervical region**	129 (45,3%)
**Lumbar region**	151 (53%)
**Cervical and lumbar region**	5 (1,7%)

*Reliability* was performed using the Intraclass Correlation Coefficient Type 2,1 (ICC_2.1_) test-retest methodology in the full sample recorded at baseline and one week (7 days) following a period of no treatment. The *sensitivity or error score* was determined from the *MDC*_*90*_ analysis that was performed as described by Stratford [25]. The standard error of the measurement (SEM) was calculated using the formula: SEM = s√(1–r), where s = the mean and standard deviation (SD) of time 1 and time 2, r = the reliability coefficient for the test and Pearson’s correlation coefficient between test and retest values. Thereafter the MDC_90_ was calculated using the formula: MDC_90_ = SEM × √2 × 1.65.

All *statistical analyses* were conducted using the Statistical Package for Social Science version 17.0 (SPSS 17.0) for Macintosh.

## Results

### Characteristics descriptive of the participants

**Figure 2:**
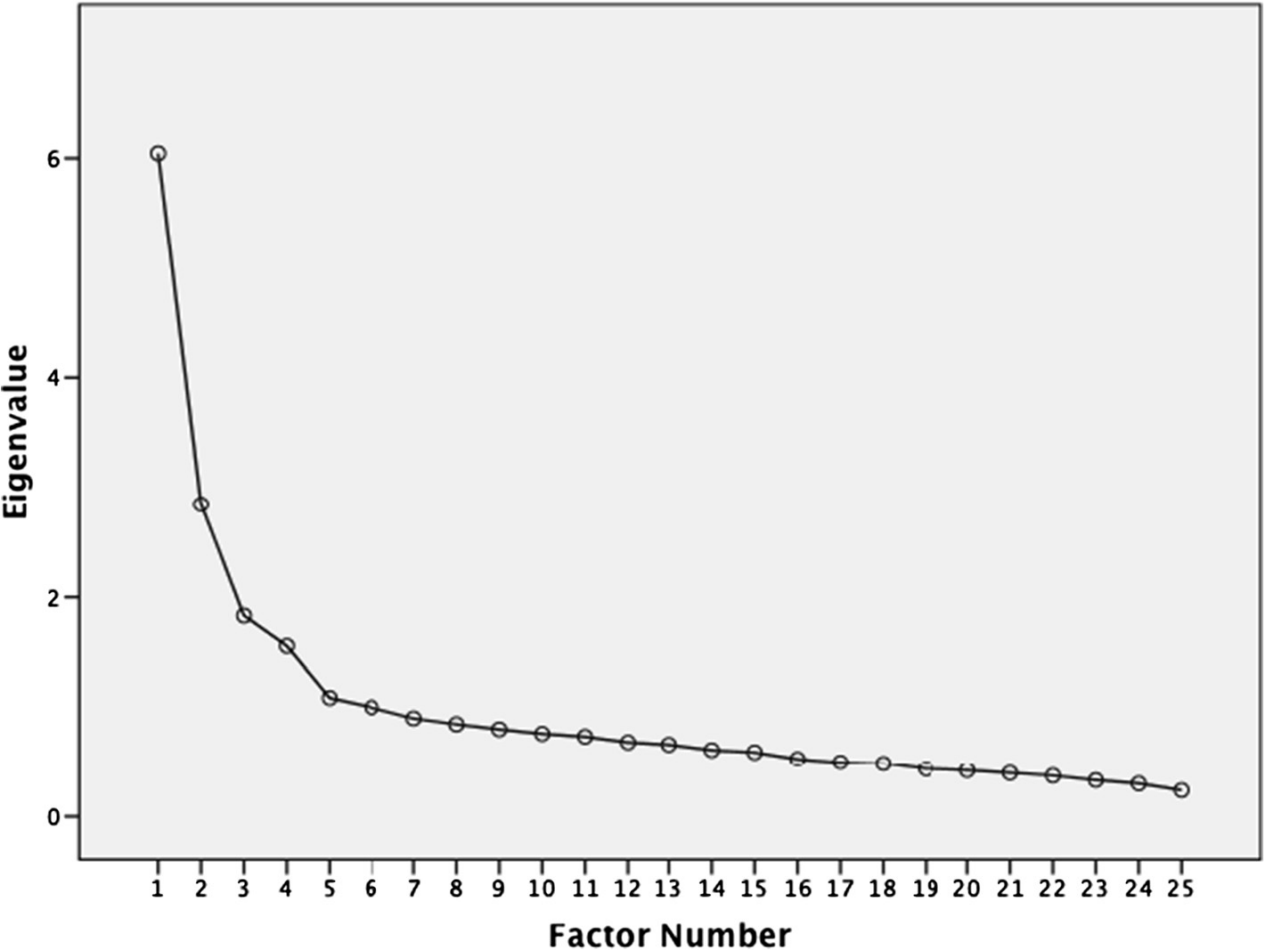
**Scree Plot indicates that an one-factor solution.**

The participants defined the major problematic region for implementation of the ODI or NDI. This provided the demographics and frequency of diagnosis for the study sample (Table 1). The SFI was translated and back translated with consideration of the Turkish cultural linguistic adaptation to provide the new SFI-Tk questionnaire without language difficulties or other conceptual misunderstanding (Figure 2). The normative mean and standard deviation values for SFI-Tk score were determined (11.9 ± 5.2 points). The SFI-Tk showed no missing responses, however the ‘half-mark’ response was only used in 11.75% of responses, but this represented 77.9% of participants. There was a high degree of *internal consistency* (α = 0.85) with an individual item α range of 0.804 to 0.882. The *test-retest reliability* was high (r = 0.93) with a noted individual range that did not exceed 0.95 (0.75 to 0.95) [23]. *Measurement error* from SEM and MDC_90_ were respectively 2.96% and 7.12%. No significant gender differences were found in the item responses.

**Figure 3:**
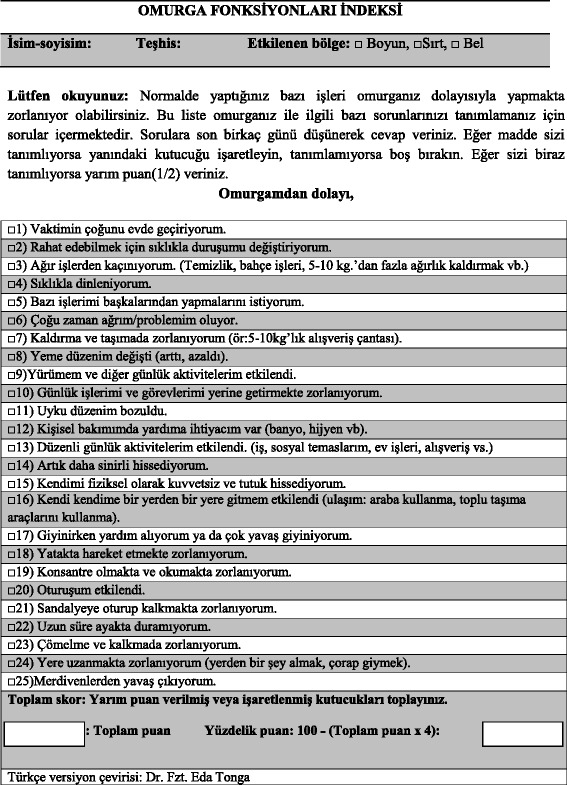
**Türkçe versiyon çevirisi.**

For *factor analysis* the correlation matrix for the SFI-Tk was determined as suitable from the Kaiser-Meyer-Oklin values (0.857) and Barlett’s Test of Sphericity (p < 0.001). This indicated that the correlation matrix was unlikely to be an identity matrix and was therefore suitable for MLE. The screeplot (see Figure 3) indicated several possible factor solutions however when all three *a-priori* criteria were accounted for a one-factor solution was determined to be optimal. The factor analysis revealed a satisfactory percentage of total variance explained by the one factor at 24.2%. It was noted that six factors had Eigenvalues >1.0 and accounted for 57.5% of variance; however those with an Eigenvalue >1.0 each accounted for <10% of variance and could be considered to be after the initial screeplot inflection point (Figure 2) and consequently were not extracted. The item loading for the one-factor solution for the MLE method and average score for each item are shown in Table 2. Criterion specific validity with ODI was high (r = 0.71, p < 0.001), with FRI and NDI it was fair (r = 0.52 and r = 0.58, respectively). For the FRI the Criterion specific validity with ODI was high (r = 0. 0,702, p < 0. 0.001), with FRI and NDI it was fair (r = 0. 601 and r = 0. 0.001, respectively).

**Table 2:** **Factor loading items for the one-factor solution and average score of items**

**Question**	**Item**	**Factor loading**	**Item average score**
1	Stay at home most of time	.447	1.94
2	Change positions frequently	.110	2.55
3	Avoid heavy jobs	.210	2.21
4	Rest more often	.409	2.22
5	Get others to do things	.471	1.67
6	Pain almost all the time	.158	2.44
7	Lifting and carrying	.222	2.56
8	Appetite affected	.273	1.46
9	Walking/normal recreation/sport	.636	2.14
10	Home/family duties and chores	.573	1.87
11	Sleep less well	.174	2.11
12	Assistance with personal care. hygiene	.639	1.54
13	Regular daily activity work/social	.550	1.93
14	More irritable/bad tempered	.208	1.85
15	Feel weaker or stiffer	.287	2.23
16	Transport independence	.664	1.60
17	I require assistance or am slower with dressing	.620	1.57
18	I have difficulty moving in bed	.534	1.93
19	I have difficulty concentrating and/or reading	.137	1.67
20	My sitting is affected	.490	2.18
21	I have difficulty getting in and out of chairs	.684	1.92
22	I only stand for short periods of time	.446	2.14
23	I have difficulty squatting and/or kneeling down	.563	2.21
24	I have trouble reaching down (e.g. pick-up things. put on socks)	.523	2.08
25	I go up stairs slower or use a rail	.566	2.24

## Discussion

### Main findings

The translation and cross-cultural adaptation of the SFI to Turkish using recognised international guidelines was achieved successfully. This provides access to a spine regional PRO instrument for Turkish speaking populations, the world’s fifth most widely spoken language. The essential psychometric properties were demonstrated and shown to be comparable to those found in the original English version and the recently translated Spanish version [30]. The adapted SFI-Tk questionnaire is self-administered and simple to use in both the clinical and research settings where spine conditions are examined and treated. The questionnaire was translated without difficulty and minimal culturally-specific examples were required. This process follows similar procedures for the cross-cultural adaptation of PRO instruments as used in studies for different scales applied in the Turkish context [22,31].

The validity, in terms of face and content, were present through the translation process and pilot testing. We choose indices that evaluated functional disability in patients with spine musculoskeletal disorders to investigate the criterion validity rather than general health measures. The criterion validity with the FRI (r = 0.52) was fair but notably lower than that (r = 0.87) found in the original study [8]. By contrast the sub-region specific criterion validity with the NDI (r = 0.58) was higher than that found in the Spanish SFI (SFI-Sp, r = 0.46). For the back regional PRO assessment the correlation was lower in this study (ODI, r = 0.71) compared to that of the SFI-Sp (RMQ r = 0.79), though this difference may be partially attributed to the different PRO that was used, ie the ODI for the SFI-Tk versus the RMQ for the SFI-Sp. It should also be noted that the RMQ is a dichotomous scale comparted to the 6 point Likert scale of the ODI. These sub-regional findings indicate a generally comparable trend with the difference in all three comparisons most likely related to the cultural and geographical differences in the populations. A further potential basis, particularly for the notably lower correlation with the FRI than found in the original study, could be the significantly lower total response rate and lower use of the ‘half-mark’ in this study (11.75% by 77.9% of participants) compared to the original study (43% by 57% of participants). This indicates that participants were aware of the application of the ‘half-mark’ option, but unlike the population in the original study it was used less often. The requirement for the patient to respond voluntarily with the half-mark may have been influenced by cultural custom and only the available option provided responded to. Whereas, if a specified option for each of the responses were provided then a higher use of the half-mark option may have resulted. A final consideration is that the FRI has not been culturally and linguistically adapted for Turkish and the version available that was used was simply translated. As such there may be aspects of the accuracy of the items that could be responsible for some of the differences in the level of correlation between the SFI and FRI Turkish versions found in this study [32].

By contrast the high reliability (r = 0.93) was comparable to the findings of both previous studies (ICC_2.1_ SFI-Sp = 0.96, SFI = 0.97). The internal consistency (α = 0.85) was identical to that of the SFI-Sp and mildly lower that the SFI (α = 0.91) confirming no item redundancy. The level of error measurement (MDC_90_ = 7.1%) was comparable to, though less sensitive than, the SFI-Sp (6.9%) and the SFI (6.4%).

The factor structure was shown as a single dimension under the *a-priori* criteria. The process of EFA is not conclusive being designed to be, as it is titled, ‘exploratory’. A confirmatory factor analysis (CFA) will be required to clarify the true status of the factor structure following the standard statistical process using the information and direction gained from EFA. The CFA requires a sample in the order of 5–10 times more than EFA [32], which was beyond the scope of this study. The exploratory nature of this analysis is illustrated by the screeplot which suggests that from 1–4 factor structures could be present as six factors had Eigenvalues above the arbitrary 1.0 cutoff. However, the *a-priori* requirements within this EFA were that multiple *a-priori* criteria must be reached [25]. Though the total variance of the first factor (24%) may be considered low within some contexts, it is still an acceptable level [24]. It was also generally 3–5 times higher than any of the other factors, none of which exceeded 10%. This variance level is also comparable to that found in the SFI-Sp (27.4%) though lower than in the SFI (33.4%). Concurrently, the interpretation of a screeplot’s inflection is highly subjective and consistently brought into question [24]. From the perspective of parsimony, the determination of a single factor is justified as the logical solution to the data analysis determined in this study [24,25]. Some of the items have low factor loadings which could be affected by cultural differences. Further research should investigate and consider the development of a short version of the SFI and the possible use of a three box dedicated response option.

In summary, the determined psychometric characteristics from this study indicated the SFI-Tk to be valid, reliable and highly suitable for use in Turkish culture. The difference in findings between this study and both the original and Spanish studies potentially suggests both cultural and geographical factors are the most likely contributors to variation. This is the reason why studies are conducted in a culturally and linguistically adapted manner [22]. A further contributor to both lower reliability and sensitivity may be related to the reduced use of the 3-point option. When this option is not utilized both values are reduced [33]. This in turn may potentially influence the factor structure by limiting the cutoff and blurring the defining lines that assist in clarifying and determining factor structure [24,25]. Consequently, modification of the SFI format to provide three separate response options per question is an option. This is as opposed to the current process of a single space where the respondent provides one of the three options. This may increase the use of the ‘half-mark’ and consequently improve reliability, sensitivity and potentially help to clarify the factor structure.

### Study limitations and strengths

The limitations to consider in this study include the sample size, particularly within the regional subgroups of neck and back. Satisfactory CFA will require a sample size in the order of 500–1000, an undertaking beyond the scope of this study. Such an analysis would ensure the factor solutions are determined that correspond to the population perspectives and also provide a stable definitive single summated score. The EFA findings from this study are from a sample size that is comparable to that used in both the original and Spanish version studies. It is also similar to most PRO studies where factor analysis is performed through EFA in a normally distributed population with the suitable MLE method and not PCA [34-38]. It was unfortunate that the reduced 3-point option use was not noted in the pilot trial as modification for the main study may have influenced the results. Further limitations include the lack of longitudinal data, specifically responsiveness, as the research was a limited duration observational study where ongoing measurements were not possible.

Strengths of the study included the standardized methods employed for all psychometric procedures and the cross-cultural adaptation process. The prospective nature and consecutive participant recruitment provided diversity in both the conditions and distribution between sub-regions. The sample size was adequate for all analyses and the reliability subgroup. Furthermore, the sample size exceeded that found in most spine PRO research where it generally does not exceed 200 and particularly in cross-cultural adaptation where 100 is often the upper limit. Importantly, this study expands both the specificity and number of instruments available for Turkish patients and health professionals.

## Conclusions

The SFI-Tk demonstrated a single factor structure providing a Turkish population specific PRO that is valid, reliable and sensitive to change. The SFI-Tk in its present form is simple to complete and easily understood. This enables it to be used in the assessment of spine musculoskeletal disorders in Turkish speaking patients. Three areas of further research are needed which include; a CFA and clarification of the factor structure, longitudinal analysis to determine responsiveness and potentially an alteration to the questionnaire’s format to provide three distinct response options per question. This latter action may potential improve the ‘half-mark’ response rate and subsequently the reliability, measurement error and possibly clarification of the factor structure. Consequently, the SFI-Tk can be recommended for clinical and research purposes in Turkish language populations.
